# Personalised Health Behaviour Support Programme in Adults With Post‐COVID Syndrome: A Randomised, Controlled Pilot Feasibility Trial

**DOI:** 10.1111/hex.70079

**Published:** 2024-10-27

**Authors:** Matthew Armstrong, Rebecca Owen, Kristen Shirley Van Niekerk, Zoe L. Saynor

**Affiliations:** ^1^ Department of Rehabilitation and Sport Sciences, Faculty of Health and Social Sciences Bournemouth University Bournemouth South West UK; ^2^ Department of Sport and Exercise Sciences, Faculty of Social Sciences and Health Durham University Durham North East UK; ^3^ School of Health Sciences, Faculty of Environmental and Life Sciences University of Southampton Southampton South West UK; ^4^ Department of Respiratory University Hospital Southampton NHS Foundation Trust Southampton South West UK; ^5^ NIHR Southampton Biomedical Research Centre Southampton Centre for Biomedical Research Southampton South West UK

**Keywords:** behaviour change, physical activity, post‐COVID syndrome, supported self‐management

## Abstract

**Background:**

We investigated whether a novel 8‐week personalised health behaviour support programme, focusing on the stability of symptoms and strategies to improve activities of daily living, was feasible and acceptable in adults with post‐COVID syndrome.

**Methods:**

In this randomised, controlled, pilot feasibility trial, 32 adults with post‐COVID syndrome (continued symptoms for ≥ 12 weeks) were randomised 1:1 to receive personalised health behaviour support (self‐reported physical activity and symptom diaries, plus seven one‐to‐one remotely delivered personalised self‐management support sessions), once weekly for 8‐weeks, or usual care (referral to online ‘your COVID‐19 recovery’ programme). The primary outcome was the feasibility of recruiting and randomising adults with post‐COVID syndrome. The secondary outcomes were to assess the acceptability and safety of the intervention and various outcome measures.

**Results:**

Of the 48 adults who expressed interest in the study, 32 (67%) were eligible and completed the baseline assessment. All 32 adults were willing to be randomised to either the personalised health behaviour support programme (*n* = 17) or usual care (*n* = 15) and 27 (age: 45 ± 12 years) adults completed follow‐up at 9 weeks. The intervention was deemed feasible, with high adherence (92% and 94% completion rates for the physical activity and symptom diaries, respectively) and excellent acceptability rates (94% ‘liked the intervention a lot’). The intervention was deemed safe, with no symptom exacerbations reported.

**Conclusion:**

An 8‐week personalised health behaviour support programme was feasible for adults with post‐COVID syndrome, with good adherence and acceptability rates. Early pilot data from this small sample also suggests meaningful improvements in physical activity, fatigue and respiratory symptoms.

**Patient or Public Contribution:**

People living with post‐COVID syndrome were involved from the outset with the study design, review of study documentation and interpretation of the data following completion. Furthermore, several participants have supported the local dissemination of findings following the completion of the study.

## 1 Introduction

Four years after the SARS‐CoV‐2 (COVID‐19) pandemic began, approximately two million adults in the United Kingdom (UK) are reportedly living with long‐term symptoms resulting from prior COVID‐19 infection [1]. Referred to as post‐COVID‐syndrome, the World Health Organisation (WHO) define this as a continuation or development of new symptoms > 3 months after initial infection [2]. Although the presentation of post‐COVID syndrome is heterogeneous and complex, for many it affects various aspects of life and can often impact their ability to be physically active [3, 4].

As the potential pathophysiological mechanisms of post‐COVID syndrome continue to evolve, multiple overlapping causes have been hypothesised [5, 6], including persisting reservoirs of COVID‐19 in tissues [7], immune dysregulation [8], microbiota dysbiosis [9, 10], inflammation [11] and endothelial dysfunction [12]. Long‐term manifestations have left many individuals with several long‐term, complex complications within neuropsychiatric, cardiovascular, respiratory, digestive, vascular and musculoskeletal systems [13]. These can affect an individual's ability to undertake activities of daily living (ADLs) and, consequently, impact quality of life (QoL). There is, therefore, a need for safe, appropriate and cost‐effective interventions to target these debilitating symptoms to regain ADLs.

Several nonpharmacological interventions, including pulmonary rehabilitation (PR) [14, 15], inspiratory muscle training (IMT) [16], pacing strategies [17, 18] and group physical and mental health rehabilitation [19], have demonstrated a variety of improvements in adults with post‐COVID syndrome. Specifically, improvements in exercise capacity, fatigue, dyspnoea and respiratory muscle strength have been reported, highlighting the range of key symptoms being investigated [14, 15, 16, 17, 19]. While these studies provide positive early evidence for potential strategies to improve key symptoms typically present and impacting the QoL of adults with post‐COVID syndrome [4], there is a need to understand how this evidence can safely be translated into people regaining ADLs [3].

Co‐developed alongside adults with post‐COVID syndrome, our personalised health behaviour support programme focuses on the stability of symptoms and strategies to sustain function, whilst not exacerbating symptoms [20]. Through regular monitoring of symptoms and PA behaviours, personalisation of strategies to sustain ADLs and correct screening for PESE, this intervention aims to support adults with post‐COVID syndrome to safely return to certain ADLs including gardening, walking to the shops and socialising with others [20].

To establish whether this is an appropriate and cost‐effective intervention to support the heterogeneous symptoms and regaining of ADLs in adults with post‐COVID syndrome, a future definitive randomised controlled trial (RCT) is required. However, an important first step is to undertake a feasibility study, to explore whether this novel programme was appropriate, adherent and acceptable for those with post‐COVID syndrome [21]. Furthermore, given the complex and often heterogeneous presentation of diverse and fluctuating symptoms typical of post‐COVID syndrome, it is important to gain insight into the safety of the developed programme to inform a subsequent definitive trial [22].

### 1.1 Study Aims

The present study aimed to evaluate the feasibility and acceptability of a novel 8‐week personalised health behaviour support programme, alongside preliminary insight into its potential effectiveness to improve key outcomes including PA, functional capacity, anxiety and depression, general health status, muscular strength and endurance in adults with post‐COVID syndrome.

### 1.2 Study Objectives

1. Assess the feasibility of recruiting and randomising adults with post‐COVID syndrome.
2. Assess the acceptability of and adherence to the 8‐week personalised health behaviour support programme.
3. Understand how safe the personalised health behaviour support programme was for adults with post‐COVID syndrome.
4. Gain preliminary insight into the effectiveness of the personalised health behaviour support programme for clinical and patient‐reported outcomes.

## 2 Methods

### 2.1 Study Design and Setting

This single‐centre, parallel two‐arm, randomised controlled pilot feasibility trial was conducted between August 2022 and March 2023. Following screening for eligibility, participants attended two face‐to‐face assessments (lasting no longer than 90 min) at baseline (week 0) and postintervention (week 9) at the Human Performance Laboratories at Bournemouth University. Eligible participants were recruited via local radio, newspapers and advertisements at community centres. The study received favourable ethics approval from the Bournemouth University Research Ethics Committee (ID: 39523) and was prospectively registered on the clinicaltrials.gov website (NCT05752331).

### 2.2 Participants

#### 2.2.1 Inclusion Criteria

Participants meeting the following criteria were included in the study (1) age ≥ 18 years; (2) willing and able to provide fully informed written consent; (3) experienced at least one self‐reported symptom of post‐COVID syndrome that impacted functional abilities for ≥ 12 weeks; (4) met at least one of the following criteria: [4a] positive SARS‐CoV‐2 Polymerase Chain Reaction antigen test (positive COVID‐19 test) during the acute phase of illness; [4b] positive SARS‐CoV‐2 antibody test at any time point and [4c] symptoms consistent with SARS‐CoV‐2 infection during the acute phase and (5) no demonstration of severe/very severe PESE/PEM after engaging in physical/mental tasks (screened using the DePaul Short‐Form Questionnaire) [23].

#### 2.2.2 Exclusion Criteria

Participants were excluded if they met any of the following criteria (1) previous admission to an intensive care unit due to COVID‐19; (2) orthopaedic, neurological or other concomitant disease that significantly impairs normal biomechanical movement patterns, as judged by the investigator at the time; (3) receiving palliative or end‐of‐life care; (4) actively participating in another research study focused on post‐COVID syndrome or (5) lacking capacity to understand the study protocol.

### 2.3 Study Procedures

#### 2.3.1 Data Collection, Randomisation and Concealment

Following screening, participants attended a baseline visit, where informed consent was obtained and physical outcome measures and paper‐based questionnaires were completed. Participants were subsequently randomised (1:1) using a computer‐generated random sequence software (randomiser.org), to receive usual care or the personalised health behaviour support programme. Randomisation was performed by a member of the research team external to the recruitment process, to avoid selection bias. Following allocation to either the intervention or usual care group, participants were provided with relevant information and a post‐assessment visit date was arranged. Concealment of the intervention allocation to participants and the study team was not possible due to the small nature of this pilot feasibility trial.

#### 2.3.2 Intervention

Participants allocated to the personalised health behaviour support programme received a package of self‐management support, which was carefully co‐designed with adults living with post‐COVID syndrome, to ensure it remained relevant to those receiving it. The support was received over the 8‐week period, comprising (1) a semi‐structured interview to outline how symptoms of post‐COVID syndrome may impact an individual's ability to conduct ADLs and learn more about their individual experiences (lasting ≤ 30 min via a secure video conferencing system or telephone); (2) seven remotely delivered one‐to‐one personalised self‐management support sessions, lasting approximately 30 min and (3) access to weekly self‐reported PA and symptom diaries, in which participants were asked to make an entry every day for 8 weeks (56 days in total). Symptoms recorded within the symptom diaries were generated by participants themselves, allowing participants to provide individualised responses. Data from the diaries was reviewed during the weekly one‐to‐one personalised self‐management support sessions, allowing for individualised feedback.

During the one‐to‐one semi‐structured interview, adults with post‐COVID syndrome randomised to the intervention shared their experiences of post‐COVID symptoms, facilitators and barriers to ADLs, as well as potential strategies to modify ADLs and manage symptom fluctuations. The focus of this discussion was to engage with participants to evoke individual internal motivations for change, encompassing both a development to change and formulating specific action plans to build upon [24, 25].

The seven remotely delivered support sessions were delivered weekly, building upon the goals and action plans formulated during the semi‐structured interview and focusing on the self‐reported data from participants' completed PA and symptom diaries the preceding week. During these sessions, a trained research assistant (training consisted of a 1 h online session with a qualified behaviour change specialist) reflected upon the diaries and provided individualised behavioural support to better manage their symptoms and understand how to manage the barriers associated with ADLs moving forward. Several behaviour change techniques were applied, including goal setting, action planning and guidance on self‐monitoring and management [26, 27].

Throughout each session, pacing strategies were closely aligned to the symptom‐contingent pacing or ‘symptom titrated PA’ approach suggested by the National Institute for Health and Care Research in the UK, which has previously been adopted as a strategy to improve energy management in ME and CFS [28, 29, 30]. Specifically, this approach encouraged participants to engage in activities guided by their perception of self‐reported symptoms using the symptom diaries provided, to avoid worsening symptoms and conserve energy levels to allow involvement in meaningful ADLs [29].

Throughout the intervention, any exacerbation of symptoms (monitored through symptom diaries) would have led to participants being asked to refrain from additional ADLs and support to focus on symptoms was provided. If symptoms continued, referral to an appropriate specialist for additional testing or intervention was provided [20]. At no point across the programme were participants encouraged to work against their symptoms to improve ADLs.

#### 2.3.3 Usual Care

Participants allocated to usual care were signposted with information about the digital self‐help available in their local area, including the ‘Your COVID recovery’ website (https://www.yourcovidrecovery.nhs.uk/) or the Long COVID physio website (https://longcovid.physio/).

### 2.4 Feasibility of Recruitment and Randomisation

The feasibility of recruiting and randomising adults with post‐COVID syndrome to an 8‐week, home‐based, remote personalised health behaviour support programme was assessed in terms of the number of adults with post‐COVID syndrome who expressed interest in participating; the number of participants screened eligible from those approached; the number of consented participants; the number of participants willing to be randomised to the personalised health support programme and the number of adults submitting all final assessment data.

### 2.5 Acceptability of and Adherence to the Intervention

Acceptability of the personalised health behaviour support programme was assessed using a bespoke questionnaire (Appendix 1), which was completed following all other week 9 assessments. The anonymised, self‐administered, questionnaire involved a selection of quantitative questions regarding the programme and the usefulness of its components (pedometer, PA and symptom diary, PA goals, face‐to‐face assessments and feedback). The opportunity to provide qualitative comments on the future useability of the programme and provide any last feedback regarding the overall project was also provided on the questionnaire.

Completeness of the self‐reported PA and symptom diaries was assessed through visual inspection, following completion of the week 9 assessment. More specifically, the overall percentage and median days completed by participants were recorded. Attendance at each of the seven remotely delivered support sessions was monitored throughout. Pedometer usage was based upon a minimum of 70 steps/day, which is in line with previous research [31, 32].

### 2.6 Safety of the Intervention

The safety of the personalised health behaviour support programme was evaluated using adverse events logs. Any adverse events (serious or otherwise) were recorded in an event log, with the type, duration, severity, date/location and whether this was related or unrelated reported.

### 2.7 Clinical Outcomes

Objectively derived PA was assessed over a 7‐day period at baseline (week 0) and following completion (week 9) of the programme. A hip‐worn accelerometer (Actigraph wGT3X, Actigraph LLC Pensacola), previously validated in people with chronic obstructive pulmonary disease (COPD) [33], was worn during wakefulness hours, with ≥ 8 h wear time per day required to be considered a valid assessment of PA [34]. Objectively derived PA from the accelerometer was reported using (1) daily steps (derived from the accumulation of step counts across a valid day of assessment); (2) movement intensity (Vector Magnitude Units [VMU], derived from the mean intensity of PA per minute over a specific period of accelerometer wear time); (3) sedentary time (defined as metabolic intensity threshold < 1.5 METS) and (4) time spent in moderate‐to‐vigorous intensity activity (defined as metabolic intensity threshold ≥ 4 METS).

Functional capacity was assessed using the incremental shuttle walk test (ISWT) and was performed in line with technical standards for respiratory disease [35]. Briefly, this test is an externally paced, incremental test that requires individuals to walk around a 10 m course, at a speed dictated by an audio recording. The test is complete when the participant is no longer able to keep up with the speed dictated by the audio recording, or requests to stop, due to symptom exacerbation or discomfort. The total number of metres each participant walked before completion is recorded. Every minute throughout the exercise and postexercise recovery, physiological measures of heart rate (HR), transcutaneous arterial oxygen saturation (SpO_2_) at the fingertip and subjective ratings of perceived exertion (RPE) were measured [35]. The 30 s sit‐to‐stand test was used, with SpO_2_ monitored throughout the exercise and postexercise recovery [36]. Peripheral muscle strength was assessed using handgrip dynamometry, with an average of three measurements on each hand taken. All physical assessments have been validated for use in individuals with chronic respiratory diseases [37].

### 2.8 Patient‐Reported Outcomes

General health status was assessed using the 5‐level EQ‐5D (EQ‐5D‐5L [38]), anxiety and depression using the Hospital Anxiety and Depression Scale (HADS [39]), fatigue using the Post‐COVID Functional Scale (PCFS) and Chalder Fatigue Scale (CFS) [40]), specific respiratory symptoms using the COPD Assessment Test (CAT [41]), breathlessness using the Medical Research Council (MRC) scale [42] and cognitive function using the Montreal Cognitive Assessment (MOCA) [43].

### 2.9 Statistical Analysis

Quantitative statistical analyses were performed using standard statistical software (SPSS version 27, IBM corporation, UK). Descriptive statistics were reported as means (standard deviations [SD]) (normal distribution) or as median [25th–75th percentiles (P25‐P75)]; skewed distribution), unless otherwise stated. Before completion of statistical analyses, the Shapiro–Wilk test was used to assess for normality, with a *p*‐value > 0.05 indicating normally distributed data. Data from the quantitative project‐tailored questionnaire was scored as categorical variables and reported as frequencies and percentages, except for the usefulness rating of the questionnaire, which was expressed as median (P25–P75).

Given that this was a feasibility study, a formal sample size was not required. With the support of a statistician, we will use the key feasibility metrics collected from this study to inform whether we will be able to recruit an adequate sample size for progression to a definitive larger trial.

Differences within groups at baseline (week 0) and completion of the study (week 9) were assessed by paired samples *t*‐test or Wilcoxon signed ranks test. Differences between groups were assessed by independent samples *t*‐tests or Mann–Whitney U test of within‐person change scores for each group. The level of significance was set at *p* < 0.05 for all statistical tests.

## 3 Results

### 3.1 Feasibility of Recruitment and Randomisation

**Figure 1:**
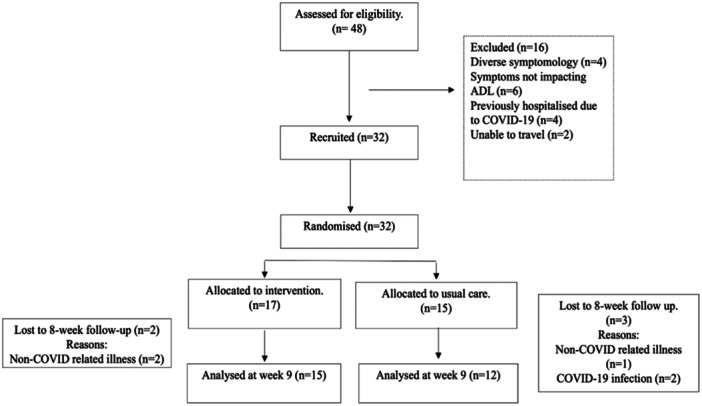
Consolidation Standards of Reporting Trials diagram of the study. N.B. COVID‐19, Coronavirus; n, number; ADL, Activities of daily living.

Overall, 48 adults with post‐COVID syndrome expressed interest in participating and were screened for eligibility. Of the 48 adults with self‐reported post‐COVID syndrome screened, 16 (33% of those screened) were not eligible to participate, due to diverse symptomology, symptoms not impacting ADLs, previously hospitalised due to COVID‐19 or being unable to travel (Figure 1). Therefore, 32 (67% of those screened) adults with post‐COVID syndrome attended a baseline assessment (Table 1) between August 2022 and March 2023, all of whom provided informed consent. All 32 adults with post‐COVID syndrome were willing to be randomised to either the intervention (*n* = 17) or usual care (*n* = 15) groups when asked before randomisation. Finally, 27 (56% of those screened) adults with post‐COVID syndrome who consented to take part completed the week 9 assessment, with dropout rates of 11% and 20% for the intervention and usual care groups, respectively. Reasons for drop‐out are provided in Figure 1.

**Table 1:** Participant baseline characteristics.

Outcome		Intervention (*n *= 17)		Usual Care (*n *= 15)	
		Mean (SD)	%	Mean (SD)	%
Age		44 (12)		46 (12)	
Sex		M = 8, F = 9		M = 7, F = 8	
Ethnic group	White British	16	94%	15	100%
	Any other white background	1	6%	0	0%
Employment status	Employment	14	82%	12	80%
	Unemployment	0	0%	2	13%
	Retired	3	18%	1	7%
COVID‐19 diagnosis criteria	Positive PCR test	1	6%	1	7%
	Symptoms consistent with COVID‐19	16	94%	14	93%
Duration of PCS symptoms (months)	< 12	7	41%	13	87%
	12‐23	10	59%	2	13%
Height (cm)		172 (11)		170 (14)	
Weight (kg)		82 (16)		83 (15)	
BMI		27 (5)		29 (5)	
FEV_1_ % PRED		85 (8)		91 (9)	
FEV_1_/FVC %		81 (11)		84 (9)	
HR (bpm)		74 (15)		73 (15)	
O_2_ Saturation (%)		98 (1)		98 (1)	
Symptoms	Breathlessness	15	88%	13	87%
	Chest tightness	4	24%	5	33%
	Pain	9	53%	6	40%
	Fatigue	15	88%	13	87%
	Impaired sleep quality	12	71%	11	73%
	Joint pain	7	41%	6	40%
	Joint swelling	3	18%	5	33%
	Limb weakness	12	71%	8	53%
	Memory loss	9	53%	14	93%
	Brain fog	16	94%	12	80%
	Cough	11	65%	10	67%
	Headache	1	6%	3	20%
	Loss of smell/taste	5	29%	6	40%
	Other	6	35%	8	53%

Abbreviations: FEV1, forced expiratory volume in 1 s; FVC, forced vital capacity; HR, heart rate; N.B. PCS, post‐coronavirus syndrome; O2, oxygen; PRED, predicted.

### 3.2 Acceptability of and Adherence to the Intervention

**Figure 2:**
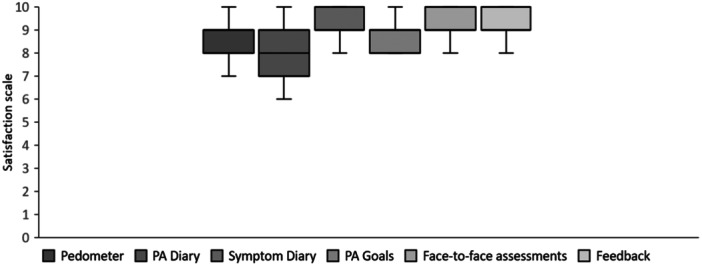
Intervention acceptability of specific components. Minimum, median, interquartile range (Q1–Q3) and maximum values are indicated. Error bars represent SD. N.B. PA, physical activity; SD, standard deviation.

The overall responses from the bespoke acceptability questionnaire are provided in Table 2. Participants who completed the intervention reported it as excellent, with 100% indicating they either ‘liked the intervention a lot’ (94%) or ‘liked the intervention’ (6%). When asked whether the intervention supported an increase in ADLs, 88% reported ‘yes, it helped a lot’, with the remaining 12% reporting ‘yes, a little bit’. Importantly, when asked to comment on the weekly increases in activity, 88% indicated they were ‘reasonable‘ and the remaining 12% reported they were ‘a little bit too low‘. Completion of PA and symptom diaries was deemed ‘very easy‘ or ‘easy‘ in all (100%) participants who completed the intervention and the useability of pedometers was also deemed ‘easy’ across its duration. The overall acceptability of intervention components (pedometer, PA/symptom diaries, step goals, activity feedback and face‐to‐face assessments) is provided in Figure 2. When asked which parts of the intervention participants would be willing to use in the future, 65% indicated the pedometer and/or PA diary, 71% indicated the telephone consultations and 94% indicated the symptom diary.

**Table 2:** Overview of participants; responses from acceptability bespoke questionnaire.

		Liked it a lot	Liked it	Neutral	I disliked it	No opinion
**Question 1)** How much did you enjoy taking part in the activity programme?		16 (94%)	1 (6%)	0	0	0
		**Yes, it helped me a lot**	**Yes, a little bit**	**Not noticeable**	**No, not at all**	**No, it rather discouraged me**
**Question 2)** Did the intervention support you to increase your physical activity to a greater level than before completing this intervention?		15 (88%)	2 (12%)	0	0	0
		**Much too low**	**A little bit to low**	**Reasonable**	**A little bit to high**	**Much to high**
**Question 3)** How did you experience the weekly increases proposed during the intervention?		0	2 (12%)	15 (88%)	0	0
		**Very easy**	**Easy**	**Not easy, but I managed**	**Difficult**	**Very difficult**
**Question 4)** How was it for you to work with the pedometer provided?		7 (41%)	10 (59%)	0	0	0
		**Very easy**	**Easy**	**Not easy, but I managed**	**Difficult**	**Very difficult**
**Question 5)** How was it for you to work with the activity and symptom diaries provided?		14 (82%)	3 (18%)	0	0	0
		**Yes, it helped me a lot**	**Yes, a little bit**	**Not noticeable**	**No, not at all**	**No, it rather discouraged me**
**Question 6)** Did you feel the telephone consultations were effective in discussing your activity and symptoms and do you think it helped to improve physical activity?		8 (47%)	6 (35%)	2 (12%)	1 (6%)	0
**Question 7)** How useful did you find the following parts of the intervention for increasing physical activity?		/10				
	The pedometer	9				
	The activity diary provided	8				
	The symptom diary provided	10				
	Daily step goals displayed on your step count diary each week	9				
	Activity feedback during each of the telephone consultations	9				
	The face‐to‐face assessment visits.	9				
**Question 8)** How often did you perform the following actions?		**Several times per day**	**Once per day**	**Sometimes, but not every day**	**Once or twice per week**	**Never**
	Look at your pedometer during the day	12 (71%)	4 (23%)	1 (6%)	0 (0%)	0 (0%)
	Look and use your activity diary	11 (65%)	5 (29%)	1 (6%)	0 (0%)	0 (0%)
	Look and use your symptom diary	16 (94%)	1 (6%)	0 (0%)	0 (0%)	0 (0%)
	Actively try to improve physical activity levels	10 (59%)	3 (18%)	3 (18%)	1 (6%)	0 (0%)
**Question 9)** Which part of the intervention would you be willing to use further in the future/recommend to future individuals in your position?		/17				
	Nothing	0 (0%)				
	The pedometer step counts	11 (65%)				
	Activity diary	11 (65%)				
	Symptom diary	16 (94%)				
	Telephone consultations	12 (71%)				
	All of the above interventions together	11 (65%)				

Completeness of the self‐reported PA and symptom diaries was high with a median (IQR) number of 54 (48–56) and 55 (50–56) recorded days over the 8 weeks, respectively. Engagement with the seven virtual self‐management support sessions was also high, with 17 (100%) participants completing sessions one to five and 15 (88%) completing sessions six to eight, with an overall median (IQR) number of 7 (7‐8) sessions attended. Over the 8‐week intervention, those completing the intervention wore the pedometer for more than 95% of days. The median wear time for the pedometer was 7 (IQR: 7‐7) days/week.

### 3.3 Safety of the Intervention

Following completion of the study, no adverse events relating to the intervention, assessment procedures or the usual care provided were reported.

### 3.4 Clinical Outcomes

Although not powered to detect change, the change from baseline to week 9 showed improvements in accelerometer step count for the intervention group (+1398 (1064) steps/day; Table 3), but not those receiving usual care (+158 (794) steps/day; Table 3), with a between‐group difference (+1240 (95% CI: 555–1926) steps/day; Table 3). For movement intensity, improvements for the intervention group (+74 (87) VMU; Table 3), but not the usual care group (+5 (72) VMU; Table 3), were also evident, with a between‐group difference (+69 (95% CI: 11–127) VMU; Table 3). Sedentary time also reduced from baseline to week 9 in the intervention group (−48 (74) mins/day; Table 3) but not those receiving usual care (−38 (127) mins/day; Table 3). Time spent undertaking moderate‐to‐vigorous physical activity (MVPA) was improved in the intervention group (+12 (11) mins/day; Table 3), but not those receiving usual care (+3 (7) mins/day; Table 3).

**Table 3:** Changes in physical activity, functional capacity and peripheral muscle strength and endurance outcome measures in the intervention and usual care groups.

Outcome	Group	Baseline (week 0)	Week 9	Within person change	Between‐group change
Daily steps (steps/day)	INT	5042 (2,022)	6440 (1,851)	1398 (1064)	1240 (555–1926)
	UC	4920 (1961)	5,078 (1,893)	158 (794)	
Movement intensity (VMU)	INT	410 (139)	484 (139)	74 (87)	69 (11–127)
	UC	416 (171)	421 (152)	5 (72)	
Time spent in sedentary activity (mins/day)	INT	580 (101)	531 (77)	−48 (74)	−10 (−83 to 64)
	UC	559 (125)	521 (162)	−38 (127)	
Time spent in MVPA (mins/day)	INT	25 (16)	37 (22)	12 (11)	9 (−29 to 47)
	UC	27 (25)	30 (23)	3 (7)	
ISWT (metres)	INT	449 (237)	518 (278)	69 (77)	21 (−39 to 78)
	UC	412 (279)	460 (277)	48 (85)	
Handgrip strength (kg)	INT	30.3 (11.9)	31.8 (12.2)	1.5 (3.0)	1.2 (0.5–3.2)
	UC	28.4 (8.7)	28.7 (9.4)	0.3 (3.4)	
30‐s STS (Repetitions)	INT	14 (4)	15 (4)	1 (1)	0 (−1 to 1)
	UC	13 (3)	14 (4)	1 (1)	

Abbreviations: ISWT, Incremental Shuttle Walk Test; KG, Kilograms; Mins, Minutes; MVPA, Moderate to Vigorous Physical Activity; N.B. INT, Intervention; STS, Sit‐to‐stand; UC, Usual Care; VMU, Vector Magnitude Units.

ISWT distance was longer at week 9 in both the intervention group (+69 (77) m; Table 3) and usual care group (+48 (85) m; Table 3), with a between‐group difference (+21 (95% CI: −39 to 78) m; Table 3). Peripheral muscle strength also improved in both the intervention (+1.5 (3.0) kg; Table 3) and usual care groups (+0.3 (3.4) kg; Table 3), with a between‐group difference (+1.2 (95% CI 0.5–3.2) kg; Table 3).

### 3.5 Patient‐Reported Outcomes

CAT scores reduced at week 9 in the intervention group (−4 (3) points; Table 4), but not those receiving usual care (0 (3) points; Table 4), with a between‐group difference (+4 points (95% CI: −6 to −1) points; Table 4). MOCA questionnaire scores increased at week 9 in the intervention group (+2 (2) points; Table 4), but not those receiving usual care (0 (2) points; Table 4).

**Table 4:** Changes in respiratory symptoms, fatigue, health status, cognitive function, breathlessness, anxiety and depression outcome measures in the intervention and usual care groups.

Outcome	Group	Baseline (week 0)	Week 9	Within person change	Between‐group change
CAT	INT	21 (5)	17 (5)	−4 (3)	−4 (−6 to −1)
	UC	18 (5)	18 (3)	0 (3)	
CFS					
Bimodal	INT	10 (2)	8 (2)	−2 (2)	−1 (−3 to 0)
	UC	9 (2)	8 (3)	−1 (3)	
Likert	INT	27 (5)	26 (8)	−1 (5)	−5 (−8 to 1)
	UC	22 (6)	26 (3)	4 (7)	
EQ‐5D‐5L	INT	0.6 (0.2)	0.6 (0.2)	0.0 (0.2)	0.1 (−0.1 to 0.3)
	UC	0.7 (0.2)	0.6 (0.2)	−0.1 (0.2)	
MOCA	INT	26 (3)	28 (2)	2 (2)	2 (0 to 3)
	UC	28 (2)	28 (2)	0 (1.9)	
MRC	INT	2 (1)	2 (1)	0 (1)	0 (0 to 1)
	UC	2 (1)	2 (1)	0 (1)	
PCFS	INT	3 (1)	2 (1)	−1 (1)	−1 (−1 to 1)
	UC	2 (1)	2 (1)	0 (1)	
HADS					
Anxiety	INT	9 (5)	8 (5)	−1 (2)	−1 (−2 to 1)
	UC	8 (4)	8 (4)	0 (2)	
Depression	INT	10 (4)	8 (3)	−2 (2)	−1 (−2 to 0)
	UC	7 (4)	6 (3)	−1 (1)	

Abbreviations: CFS, Chalder Fatigue Scale; HADS, Hospital Anxiety and Depression Scale; INT, Intervention; MOCA, Montreal Cognitive Assessment; MRC, Medical Research Council; N.B: CAT, COPD Assessment Test; PCFS, Post COVID Functional Scale; UC, Usual Care.

### 3.6 Self‐Reported Diaries

Improvements in pedometer step counts were reported at week 9 (+1567 (2947) steps/day). The average number of minutes completing ADLs improved at week 9 (+18 (6) minutes), with the most common ADLs including ‘walking the dog’, ‘taking a walk around the park’ and ‘gardening’.

The most commonly reported symptoms across the 8‐week intervention were fatigue (82%), breathlessness (71%), perceived lactic acid (24%), brain fog (65%) and migraine (65%). Other self‐reported symptoms included aching muscles, dizziness, memory loss, restlessness and low mood. Changes in self‐reported symptoms (Likert scale [1 = no symptom to 10 = extremely severe symptoms]) at week 9 included reductions in symptoms of fatigue (−2 (2) units), perceived lactic acid (−2 (1) units), breathlessness (−2 (2) units), sleep disturbance (−4 (2) units) and migraine (−1 (2) units).

## 4 Discussion

This randomised, controlled, pilot feasibility trial, co‐developed with adults living with post‐COVID syndrome evaluated the feasibility and acceptability of a novel 8‐week, home‐based, remote personalised health behaviour support programme, which aims to support adults with post‐COVID syndrome to safely return to certain ADLs. Principal findings were that the programme appeared feasible, safe and acceptable for improving ADLs in this population and may have the potential to reduce key symptoms of post‐COVID syndrome, including breathlessness, fatigue and brain fog.

The primary outcome was the feasibility of recruiting and randomising adults with post‐COVID syndrome for the current study. The number of adults with post‐COVID syndrome who expressed interest in participating was high and aligned with other published studies focusing on nonpharmacological interventions in adults with post‐COVID syndrome, including physical training [14, 15], pacing strategies [17, 18] and breathing techniques [16]. The number of participants screened eligible for the study who provided informed consent and achieved the required criteria to progress to a definitive RCT (recruitment of > 30% of eligible participants [44]. Retention rates across both study arms were high (≥ 80%) and aligned with previous literature stating that retention ≥ 80% is unlikely to threaten the overall validity of research trials [45].

Intervention acceptability was high with ≥ 90% overall completeness for the self‐reported diaries and attendance at the remote self‐management support sessions. Utilisation of these diaries/sessions has the potential to provide adults with post‐COVID syndrome with the capacity to observe, self‐manage and tailor their ADLs to manage and reduce the likelihood of exacerbating symptoms. This was evident in the current study with improvements in objectively derived PA supported by reductions in self‐reported symptoms of breathlessness, fatigue and brain fog.

The above paragraph aligns closely with guidelines on the clinical management of COVID‐19 from the WHO [46]. Within this document, the safe return to participation in ADLs requires a tailored package of education and skills training on techniques for managing energy conservation. Several techniques presented by the WHO aligned closely with the current study, with the seven remote self‐management sessions offering a variety of support tools including planning activities, tailoring tasks and incorporating rest through self‐monitoring. The WHO have suggested that providing individuals with the ability to track and monitor symptoms, using PA and symptom diaries, was an important consideration [46]. This was emphasised throughout the current study with high completeness of self‐reported PA and symptom diaries reporting improvements in self‐reported PA and key symptoms of post‐COVID syndrome. Therefore, future research must incorporate these techniques as an integral component in the care of adults with post‐COVID syndrome, to avoid symptom exacerbation, relapse and loss of confidence [47].

Given the complex and often heterogeneous presentation of symptoms typical of other post‐viral syndromes during physical exertion, the monitoring and management of fluctuating symptom severity and PESE during this study was important [48]. With support from local focus groups, ME/CFS and the ‘Long COVID physio’ literature, symptom‐contingent pacing was utilised throughout the intervention presented in this pilot feasibility trial [29, 30]. Unlike graded exercise therapy or fixed activity increments, utilisation of symptom‐contingent pacing has provided opportunities for participants to continuously monitor ADLs and adjust according to individualised symptoms [29, 46]. The use of symptom‐contingent pacing could be a major reason why improvements in objective PA were supported by reductions in self‐reported symptoms in the current study. Similar approaches in adults with CFS have shown effective improvements in fatigue, mental health and perceived exertion [29, 49]. However, these studies have not combined improvements in symptoms with an analysis of objective PA. Therefore, to our knowledge, this pilot feasibility trial is the first of its kind to investigate the impact of symptom‐contingent pacing on objectively measured PA and symptom management collectively in adults with post‐COVID syndrome.

Several published studies have focused on similar interventions in adults with post‐COVID syndrome, including physical training [14, 15], pacing strategies [17, 18] and breathing techniques [16]. All of these studies have provided vital findings to support adults with post‐COVID syndrome, including improvements in fatigue, overall health status and perceived breathlessness. However, numerous distinctions that emphasise the importance of the current study should be highlighted.

Firstly, none of the aforementioned studies provide any route towards translating improved symptoms into regaining ADLs. It is perceived that the novel approach taken in this study will allow the safe return to certain ADLs without the risk of exacerbating symptoms [20]. Secondly, none of these studies have objectively measured PA, meaning they were unable to provide an accurate indication of the type, duration, frequency or intensity of PA delivered during their interventions [17, 18]. Through objectively measuring PA in the current study, we have ensured that adults with post‐COVID syndrome are safely remaining within their tolerance thresholds, reducing the risk of exacerbating symptoms [48].

In a recently published protocol, the effectiveness of a personalised self‐management intervention for adults with post‐COVID syndrome (The Listen RCT) aims to provide six one‐to‐one personalised sessions and monitoring tools to help manage long‐term symptoms [50]. It is hoped that this approach will integrate the regaining of ADLs as a key component of the intervention to further investigate these tools in adults with post‐COVID syndrome moving forward.

### 4.1 Study Implications and Future Directions

Recent data from the Office for National Statistics has reported an increased prevalence of post‐COVID syndrome across the UK, meaning approximately two million people continue to live with the long‐term consequences of this condition [1]. While usual care provides some benefits, through its multidisciplinary approach, it fails to appreciate the heterogeneous nature of post‐COVID syndrome [4], which requires more complex behaviour change interventions to regain certain ADLs.

This study provides promising initial evidence that a more complex, 8‐week, personalised intervention, co‐developed with adults living with post‐COVID syndrome to support behaviour change to regain certain ADLs, appears to be feasible, acceptable and safe. A larger, definitive RCT is therefore now warranted, to explore the clinical and cost‐effectiveness of delivering this intervention across multiple sites.

### 4.2 Study Limitations

Several limitations should be considered following the completion of this study. Firstly, self‐reported diaries were not provided to the usual care group, due to the stimulus and incentive it may have provided this group. This has several implications, including a lack of ability to blind the research team to the study allocation, and the inability to explore the natural recovery of the usual care group. Secondly, the use of self‐reported diaries may also provide a potential source of bias due to the subjectivity of the measures. Thirdly, we acknowledge the lack of diversity within the study sample, making it difficult to generalise findings to the whole heterogeneous population of post‐COVID syndrome. While more research is needed on more diverse post‐COVID populations, we will seek more variation in a future trial.

## 5 Conclusion

In conclusion, this randomised controlled pilot feasibility trial has established that an 8‐week personalised health support programme, aiming to improve the ability of adults with post‐COVID syndrome to complete more ADLs and reduce self‐reported symptoms, was safe, feasible and acceptable. Furthermore, when compared to usual care, the personalised health support programme improved PA and respiratory symptoms, which were supported by reductions in self‐reported symptoms of fatigue, lactic acid, breathlessness, sleep disturbance and migraines. This study therefore supports progression to a definitive larger trial to further explore the clinical and cost‐effectiveness of delivering this intervention across multiple sites.

## Author Contributions

**Matthew Armstrong:** conceptualisation, methodology, investigation, writing–original draft, writing–review and editing, validation, visualisation, formal analysis, project administration, data curation, supervision, resources. **Rebecca Owen:** methodology, writing–original draft, writing–review and editing, formal analysis, project administration, data curation. **Kristen Shirley Van Niekerk:** formal analysis, project administration, data curation, investigation. **Zoe L. Saynor:** writing–original draft, writing–review and editing, supervision, resources, project administration, formal analysis.

## Conflicts of Interest

The authors declare no conflicts of interest.

## Supporting information

Supporting information.

## Data Availability

The data sets used and analysed during the current study are available from the corresponding author upon reasonable request.
