# Supplementary material for: Personalised Health Behaviour Support Programme in Adults With Post‐COVID Syndrome: A Randomised, Controlled Pilot Feasibility Trial
Source: Health Expect. 2024 Oct 27;27(5):e70079. doi: 10.1111/hex.70079 (PMC11513405; doi:10.1111/hex.70079)
Supplement: Supplementary file 1 — Supporting information. [file HEX-27-e70079-s001.docx]

**The effectiveness of personalised health behaviour tips and pacing to support activities of daily living in those with long term symptoms of COVID-19**

**Patient satisfaction form**

**Participant ID:** Click or tap here to enter text.

**Completion Date:** Click or tap to enter a date.

How much did you **enjoy taking part** in this program?

- I liked it a lot
- I liked it
- Neutral
- I did not like it
- No opinion

***Explain why?*** Click or tap here to enter text.

Did the intervention **support you in increasing** your physical activity to a greater level then prior to completing this intervention?

- Yes, it helped me a lot
- Yes, a little bit
- Not noticeable
- No, not at all
- No, it rather discouraged me

***Explain why?*** Click or tap here to enter text.

How did you **experience the weekly increases** proposed during the intervention?

- Much too low
- A little bit too low
- Reasonable
- A little bit to high
- Much too high

***Explain why?*** Click or tap here to enter text.

How was it for you to work with a **pedometer** on a daily basis?

- Very easy
- Easy
- Not easy, but I managed
- Difficult
- Very difficult

Explain why? Click or tap here to enter text.

How was it for you to work with the **activity and symptom diaries** provided?

- Very easy
- Easy
- Not easy, but I managed
- Difficult
- Very difficult

Explain why? Click or tap here to enter text.

Did you feel the **telephone consultations** were effective to discuss your activity and symptoms and do you think it helped to improve physical activity?

- Yes, it helped me a lot
- Yes, a little bit
- Not noticeable
- No, not at all
- No, it rather discouraged me

Explain why? Click or tap here to enter text.

Please let us know your thoughts on travelling onto the university campus to conduct two face-to-face assessment sessions.

Click or tap here to enter text.

**How useful did you find the following parts of the intervention for increasing physical activity? (please score out of 10)**

1. The **pedometer** /10
2. The **activity diary** provided /10
3. The **symptoms diary** provided /10
4. Daily **step goals** displayed on your step count diary each week /10
5. Activity **feedback** during each of the telephone consultations /10
6. The face-to-face assessment visits /10

How often did you (in general) perform the following actions?

|  | Several times per day | Once per day | Sometimes, but not every day | Once or twice per week | Never |
| --- | --- | --- | --- | --- | --- |
| Look at your pedometer during the day |  |  |  |  |  |
| Look and use your activity diary |  |  |  |  |  |
| Look and use your symptom diary |  |  |  |  |  |
| Actively try to improve physical activity levels |  |  |  |  |  |

Which part of the intervention would you be willing to use further in the future/recommend to future individuals in your position?

- Nothing
- The pedometer step counts
- Activity diary
- Symptom diary
- Telephone consultations
- All of the above interventions together

What component of the intervention would you like to change in the future?

Explain:

Click or tap here to enter text.

Would you like to add a comment?

Click or tap here to enter text.

Thank you for your time throughout this research study and I hope the benefits you have received taking part will continue!
